# Neurotree: A Collaborative, Graphical Database of the Academic Genealogy of Neuroscience

**DOI:** 10.1371/journal.pone.0046608

**Published:** 2012-10-05

**Authors:** Stephen V. David, Benjamin Y. Hayden

**Affiliations:** 1 Oregon Hearing Research Center, Oregon Health and Science University, Portland, Oregon, United States of America; 2 Department of Brain and Cognitive Sciences, University of Rochester, Rochester, New York, United States of America; Northwestern University, United States of America

## Abstract

Neurotree is an online database that documents the lineage of academic mentorship in neuroscience. Modeled on the tree format typically used to describe biological genealogies, the Neurotree web site provides a concise summary of the intellectual history of neuroscience and relationships between individuals in the current neuroscience community. The contents of the database are entirely crowd-sourced: any internet user can add information about researchers and the connections between them. As of July 2012, Neurotree has collected information from 10,000 users about 35,000 researchers and 50,000 mentor relationships, and continues to grow. The present report serves to highlight the utility of Neurotree as a resource for academic research and to summarize some basic analysis of its data. The tree structure of the database permits a variety of graphical analyses. We find that the connectivity and graphical distance between researchers entered into Neurotree early has stabilized and thus appears to be mostly complete. The connectivity of more recent entries continues to mature. A ranking of researcher fecundity based on their mentorship reveals a sustained period of influential researchers from 1850–1950, with the most influential individuals active at the later end of that period. Finally, a clustering analysis reveals that some subfields of neuroscience are reflected in tightly interconnected mentor-trainee groups.

## Introduction

Neuroscience is a highly interdisciplinary field that draws researchers from a variety of backgrounds ranging across the sciences and humanities. Understanding how ideas are drawn into neuroscience from other fields and how they interact is of central interest to the history of science. Given the large size of the field (the annual meeting of the Society for Neuroscience regularly draws over 30,000 attendees), it is becoming increasingly difficult even for active neuroscientists to simply observe and describe the trends governing the field. These problems are ripe for computational tools that enable systematic organization and study of large data sets containing information about individual neuroscience researchers.

An academic mentorship database provides several additional benefits to a research community, allowing new members to learn the lay of the land and to place themselves within the context of their field. Several fields of science have published their own mentorship history in some form or another, including mathematics, computer science, primatology and physics [1]–[3]. Analysis of academic genealogies has provided useful insight into training environments that produce the most productive researchers in their later careers [4].

This report describes Neurotree [5], an online database that documents mentor relationships within the field of neuroscience and with scientists in related fields. Information about mentorship is presented in an intuitive family tree format that enables straightforward visualization and navigation of the database [3]. Data in Neurotree have been provided by several thousand volunteer users since the site went live in 2005, and the database continues to grow daily. In addition to traditional neuroscientists, Neurotree contains information about physiologists, philosophers, physicists, computer scientists, economists and others who have either trained neuroscientists or performed neuroscience research themselves. Some users have expanded the historical reach of the database, allowing the majority of researchers in the database to trace their mentorship in several chains back to the earliest days of the University in the twelfth century or earlier.

The dataset contained in Neurotree provides a valuable resource for quantitative study of the individuals and disciplines that have influenced neuroscience throughout its development. Because mentors often train multiple students, understanding academic mentorship also allows one to follow the divergence of theories and techniques through different descending branches of the tree. Here we describe the data that constitutes Neurotree, assess how completely and accurately it documents mentorships, and illustrate how it can be used to understand large-scale trends in the field of neuroscience.

## Methods

**Figure 1:**
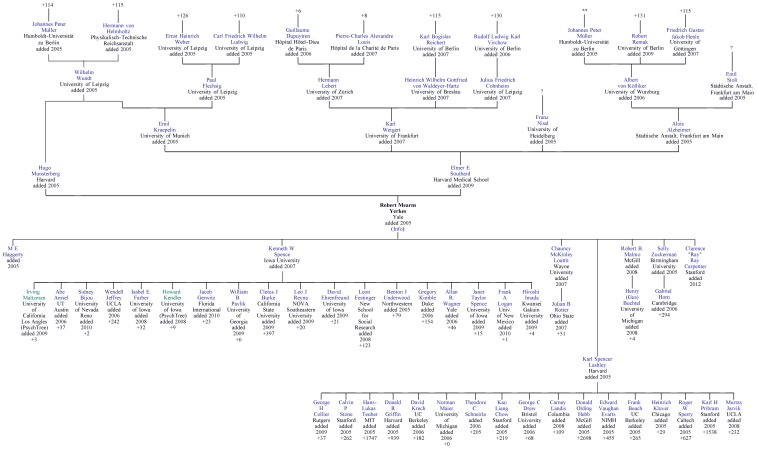
Example mentorship diagram. This plot shows a typical mentorship tree diagram for one researcher in Neurotree, Robert Yerkes, modified to fit in print format and to display extra details from the database. The tree mimics the style of biological family trees, in which the central node is linked downward to children (trainees) and up to parents (mentors). Each node in this graph is annotated with the year in which it was added to Neurotree (e.g., “added 2006”), illustrating how the tree has filled in over time. Numbers (e.g., “+37”) on nodes at the top and bottom of the tree indicate the number of ancestors and descendents, respectively, from that node.

**Figure 2:**
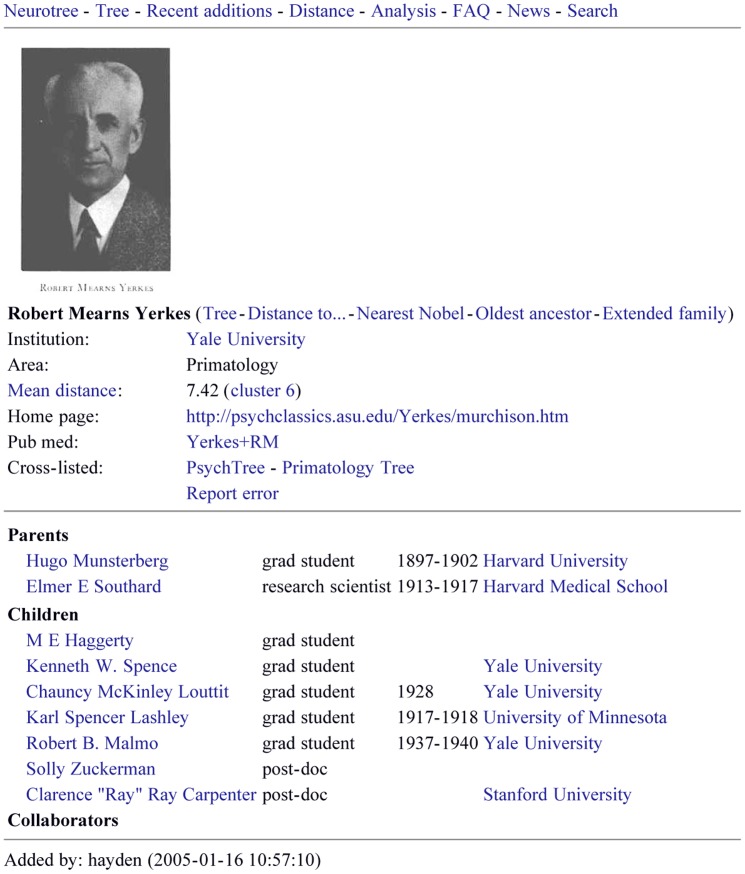
Example biographical information page. Information about each researcher in Neurotree can also be displayed in a more detailed format, as in the example of Robert Yerkes shown here. Detailed information, when available, includes a photo or drawing, links out to various analyses on the Neurotree site, biographical notes and a link to a relevant off-site web page. In addition, dates and locations of mentor relationships are provided when available.

Neurotree is accessed through a public website at http://neurotree.org/ [5]. The site is built using a set of custom-programmed scripts that present information about mentor relationships between neuroscience researchers and allow site visitors to edit and add to that information. Mentor relationships are presented graphically in a tree format (Figure 1) and in a more detailed biographical format (Figure 2). In addition to the database itself, Neurotree contains a search feature, a FAQ, a discussion board, and dynamically updated analyses of the database contents. As a service to the neuroscience community, we have made the data in Neurotree available to interested researchers (see below).

### Database architecture

**Figure 3:**
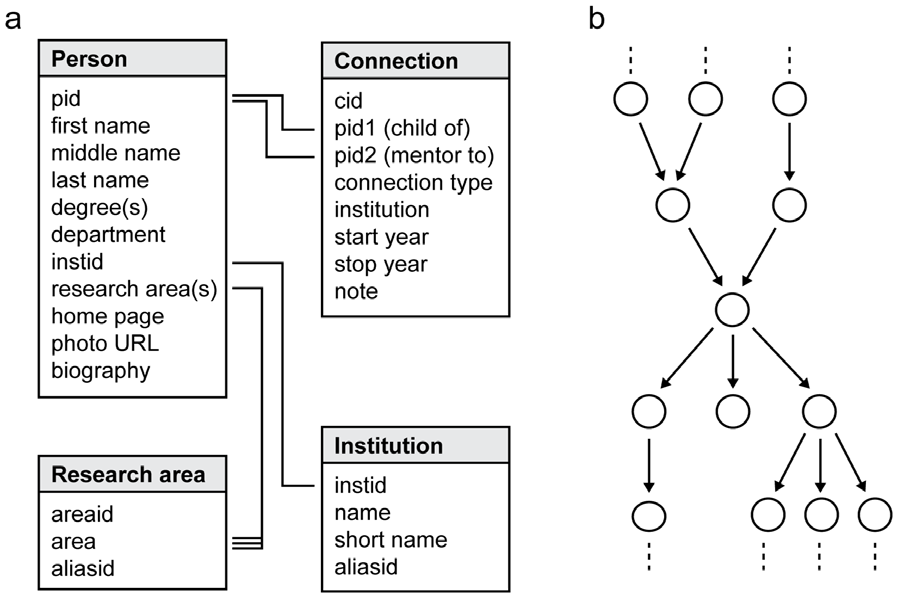
Architecture of Neurotree. **A,** Mentorship information is stored in a relational database with two core tables. The *person* table contains information about individual researchers, identified by a unique pid. The *connection* table contains information about the relationship between two people in which pid1was trained by pid2. Supplementary tables index information about institutions and research areas associated with researchers. **B,** The mentor relationships in Neurotree can be described as a directional tree in which an arrow connects a mentor to a trainee. In nearly all cases, the graph flows in a single direction without loops. In the case of collaboration, the relationship may be bi-directional. Collaborations currently represent a very small portion of the data in Neurotree.

The core of Neurotree is a relational database consisting of two main tables (Figure 3A). Each row in the *person* table contains information about an individual researcher (name, most recent institution, research areas), identified by a numerical index, *pid*. Mentorship relationships between two people are then recorded in the *connection* table. Each row of the connection table links to two nodes by *pid1* and *pid2*. The nature of the relationship is identified by a *connection type*. For example, a connection type of 1 indicates that node pid1 was a graduate student of pid2. Implicit in the relationship code is directionality, indicating that pid1 was the trainee of pid2 (Figure 3B). The types of relationship are detailed in Table 1.

**Table 1:** Connection types (stored in the connection table, Figure 3A) defining mentor relationships between pairs of researchers in Neurotree.

Connection type	Description
0	Research assistant. Undergraduate, pre-bachelor's degree.
1	Graduate student. Work lead to master's or doctoral dissertation.
2	Postdoctoral fellow. Short-term employment after earning doctorate.
3	Research scientist. Long-term employment after doctorate.
4	Collaborator. Non-directional, work together influenced each other's thinking.

Additional database tables are designed to link researchers to a set of institutions and research areas. We have imposed no restrictions on the contents of these auxiliary tables. Thus if a new institution or research area is entered by a user, this will result in the addition of a new entry to the respective table.

The simple architecture of the tree reflects an attempt to incorporate information into the Neurotree database using an organic, unrestricted approach. The field defining an individual's research area is not restricted to a fixed set of terms and, instead, can be whatever the user adding data to the site considers appropriate. This approach leads to an obvious potential for lack of consistency, but at the same time permits a very flexible and dynamic catalog of research areas in neuroscience, which evolve rapidly. Most importantly, the flexible structure of the Neurotree database means that future researchers can amend, improve, and augment this structure in the future.

### Ambiguities in cataloging an academic discipline

As it has grown, Neurotree has been confronted with ambiguities over the precise definition of mentoring relationships, relying on modern terms such as “research assistant” and “postdoctoral fellow.” Such designations have evolved over the course of history, and differences persist between countries today. In addition, many influential individual careers have taken idiosyncratic paths that do not reduce easily to a simple set of relationships. Our preference is to be pragmatic and suggest that contributors use the term that seems most appropriate based on the stage of an individual's career and to document all relationships that substantially influenced the trainee's work (see Table 1).

Another technical problem is that some scientists have multiple home institutions. We have adopted a policy that the most recent home institution should be the official institution. We acknowledge that this leads to some confusion, as when a scientist is closely associated with one location and then, near retirement, moves to another one. We plan to revise the database structure to track institutional affiliation over time.

### Reliability of Neurotree

Like Wikipedia and other crowd-sourced projects, Neurotree is publicly editable, and, as a consequence, is not guaranteed to be accurate. Formal documentation is not required for submissions, but we have implemented a simple reporting system for flagging and resolving possible errors. Error reports can be submitted by any site visitor. A volunteer group of editors validates these reports and makes appropriate changes to the database. Generally error reports can be checked against information publically available on the Internet. If the need arises for a more extensive discussion, editors may choose to contact the individuals who entered the information in question or to open the discussion with other editors. In the case of discrepancies that cannot be definitively resolved (typically in the case of historical figures whose biographies may be incomplete), the information in the tree is labeled as potentially unreliable.

In addition to inaccuracies in data, an additional issue is that information in the database may not be complete, as only a subset of mentors or trainees may be listed for any given individual. In this study, we explore how several statistical properties of the tree have evolved over time in order to understand how complete information is in the current tree.

### Graphical analysis of the Neurotree database

Neurotree can be described as a graph composed of nodes (researchers) and directional edges (mentor relationships, Figure 3B). This very simple model permits a number of analytical approaches based on graph theory. Here we describe examples of analysis that can be applied to the data. The results described in this study are based on the contents of the Neurotree database as of March 31, 2012, 87 months after the database was established.

#### Distance metrics

The distance, *d*(*a*,*b*) between researchers *a* and *b* can be measured as the smallest number of edges between nodes, either in a signed direction (e.g., mentor to trainee) or independent of direction. Most nodes are connected in a single graph, but if a connection does not exist, then the distance is defined as infinite. In order to factor in infinite connection distances, the average distance between one node and *N* other nodes is defined as the mean of reciprocals,
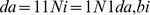
For infinite *d*, the reciprocal is defined as zero and permits a numerical solution for average distance.

#### Fecundity

In order to characterize how prolific one individual has been in training researchers who have themselves been productive, it is possible to count offspring using the directional information in the graph. A mentorship tree is defined as the graph of nodes along the mentor-to-trainee axis from an individual researcher. In this way, the total impact, *I*, with *n*_1_ trainees, can be measured recursively by traveling down the mentorship tree,
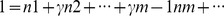
where *n*_2_ is the total number of trainees of the *n*_1_ trainees, and *n_m_* is the number of trainees stepping down through *m* successive generations. In the case that the normalization factor, *γ*, is 1, this simply reduces to counting the total number of offspring. For *γ* = 1/2, trainee counts from subsequent generations are weighted by one-half, allowing for a more balanced comparison between researchers at different points in the past. Mentorship trees are often interconnected (i.e., in biological terms, incestuous), and individuals may appear multiple times in a single tree. To avoid bias from such repeats, individuals are counted only once, at the point of closest proximity to *a*.

An alternative metric has been proposed for studying fecundity based only on the researchers trained directly by a mentor rather than iteratively across generations [4], equivalent to *γ* = 0 here. Both metrics are helpful for understanding the impact of a researcher on a field. The iterative statistic used here is specifically helpful as a measure of long-term impact on the field, as it accounts for whether the mentorship was effective enough to produce trainees with a high impact of their own.

#### Clustering

Given that neuroscience, like any academic discipline, contains a number of sub-fields, one might expect clustering, in which researchers tend to train others who continue work within their subfield rather than in a new, completely unrelated field. We studied this problem by clustering the Neurotree database according to mentorship relationships. A sparse connection matrix was defined, *C*(*a*,*b*), with a value of 1 when a relationship existed between researchers *a* and *b*, and a value of 0 otherwise. The matrix was divided into 60 partitions using spectral factorization, an effective algorithm for clustering large, sparse data sets [6]. This procedure computed the 60 largest eigenvectors of the matrix and then applied *k*-means clustering (cosine distance) to the projection of *C* into the eigenvector space. The *k*-means algorithm produces clusters with minimum distance between each researcher and the centroid of their cluster in the eigenvector subspace.

Clusters were assigned numbers based roughly on chronology, ordered by the average generation of each group. In order to characterize their basic features, each cluster was labeled with two representative researchers (the two individuals with shortest distance to other members of the cluster) and two representative research areas (the most common research areas across all members of the cluster). The clusters were plotted using open source software (Graphviz, [7]). An interactive version of the cluster map is available on the Neurotree web site.

The single parameter required by the spectral factorization algorithm, cluster count, was not identified by objective criteria. The value of 60 produces a number of clusters that could be plotted on a single graph and which demonstrated the variable topology of clusters identified by spectral factorization (large versus small, tightly versus loosely connected, etc., see Results). Changing the number of clusters over a range of 40–80 did not have a major impact on the patterns observed in these properties.

It is likely that other, more advanced clustering algorithms may provide cleaner and more interpretable results. We chose the spectral factorization method for this study as a compromise between a more standard *k*-means analysis and several more complex possibilities. The *k*-means algorithm was unable to converge to a stable solution, given the sparse structure of the connection matrix. More complex algorithms may prove effective at elucidating important clusters. However, differences between such algorithms effectively represent hypotheses about the structure of the mentorship network. The comparison of different clustering algorithms is an important problem in itself that should be addressed in future studies. The analysis in this study provides a demonstration that clustering can reveal structure in the Neurotree graph.

#### Tracking tree features over time

Neuroscience is an evolving field, and many features of the graph are likely also to evolve over time. Thus to understand the tree, it can be helpful to measure statistics as a function of the time at which researchers performed their work. The database has a capacity for logging the dates of mentor relationships, but this information is often incomplete. As an alternative to using absolute dates, we labeled each researcher with their *generation*, which counted the minimum number of steps from one individual back to their oldest ancestor. The majority of researchers in the tree could trace their mentorship directly back to a single individual (Florentius Radewyn). Thus we could align researchers in this group along a single temporal axis. The analysis of historical dynamics focused on this subset of the Neurotree database.

### Citations and data export

Data contained in Neurotree are available for export under the Creative Commons License 3.0. The data may be used freely by other researchers, and publications using the data should cite this publication as a source. Instructions for requesting the data are included in the site FAQ at http://neurotree.org/neurotree/faq.php [5].

### Ethics statement

Data in Neurotree are collected from publicly available web sites and databases. Thus this study represents an analysis of information in the public domain. In order to respect potential privacy concerns, we have given individuals the opportunity to have their information removed from the Neurotree simply by submitting an error report or contacting the site administrators.

## Results

### Neurotree's seeding and growth

Neurotree [5] was born out of the authors' attempt to map out the mentoring relationships in the subfield of visual systems neuroscience. Although its original form was a large piece of paper, the problem turned out to be too complex for paper and was translated in 2005 into a relational database (Figure 3A) that could be displayed dynamically in a family tree format through a set of PHP scripts (Figure 1). Because the information was thought to be of interest to a broader community, the initial database of several hundred researchers was made publicly accessible online, with an interface for adding data.

As the site was indexed by search engines and subsequently discovered by researchers with related interests, the scope of the database grew unexpectedly beyond its original focus. An example illustrates how the tree filled in around one researcher in the database, Robert Yerkes, a comparative psychologist (Figure 1). As is typical for neuroscience, the number of trainees is larger than the number of mentors, reflecting the expansion of the field in recent decades. More detailed information can also be displayed in a biographical format that includes links to additional information on the Internet and dates of mentor relationships, when this information is available (Figure 2).

The site has also taken on a number of new functions in addition to its original design as an education resource. Neurotree can serve as a tool for disambiguating between researchers with the same name, a problem that occurs frequently in such a large field. It is also used as a professional networking tool, enabling journal editors, employers and potential collaborators to learn more about individuals they encounter in the community.

As the site has grown, we have taken a broad view of the term “neuroscience”, and have chosen to err on the side of inclusiveness. Neuroscience has been and continues to be a highly interdisciplinary field, and maintaining information about the relationship between neuroscience and related fields is valuable in and of itself. Thus we have deliberately encouraged people to submit information about connections between neuroscientists and well-known individuals in other fields. This information provides insight into connections across a broader academic community and with the historical roots of the field.

**Figure 4:**
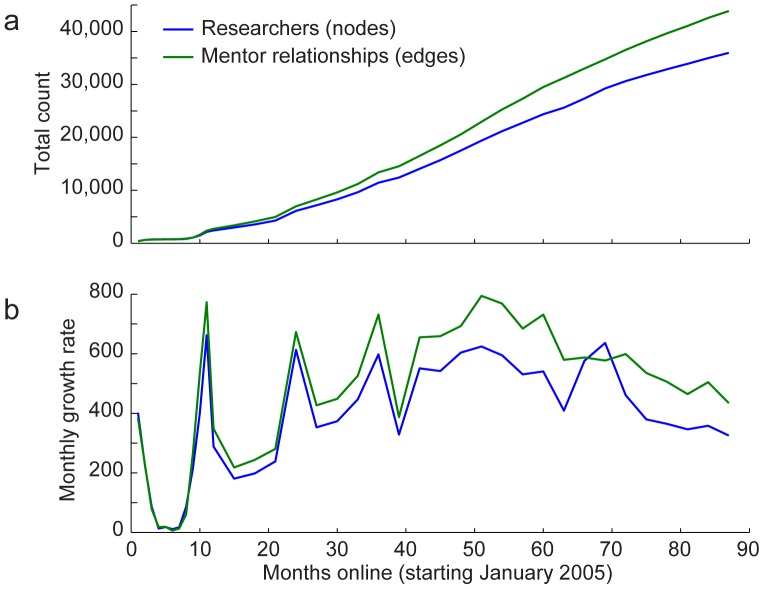
Five years of Neurotree's growth. **A,** Curves show the monthly total of researchers (blue) and mentor relationships (green) recorded in Neurotree since it was established in January 2005. **B,** Average monthly growth of Neurotree, plotted as in A. Growth has slowed from the initial rise and currently averages 300–400 new researchers per month.

The site continues to grow and, as of January 2012, draws an average of 25,000 unique visitors each month. The original database was seeded with about 500 researchers and has since grown to 35,000, with about 300 added each month (Figure 4).

### Basic graphical properties

One of the benefits of any genealogical database is the ability to map the connections linking members of the tree. Neurotree can be described mathematically as a graph, with nodes (researchers) connected by edges (mentor relationships, Figure 3B). Currently, 30055/35953 (84%) of researchers in the tree are linked in a single large graph, and 22970 of those researchers (64%) can trace their academic roots directly back to a single ancestor (Florentius Radewyn of Deventer, Holland). As discussed in the Methods, it is sometimes difficult to make an exact designation of neuroscientist versus philosopher. Regardless of his field of study, a clear line of mentorship can be drawn to this individual (and back many more generations in other fields). The difference between the total number of linked researchers versus the number of direct descendents reflects individuals who are linked to the main graph as mentors (arrows going down, Figure 3B), but who themselves do not currently have a record of their mentorship linking back to the main graph (arrows arriving from above).

The graphical structure of Neurotree permits a number of analyses, some of which we demonstrate below. Because the database depends on contributions of volunteer users, however, the results of any analysis must be interpreted with the caveat that information in the database is not complete. As the tree matures and fills in, we expect the data to become increasingly more reliable.

### Accuracy and completeness of the Neurotree database

In order to assess the reliability of the current database, we performed simple spot checks on its content. First we examined the accuracy of 100 randomly selected researchers in Neurotree compared to information available elsewhere on the Internet. Of these, 72 were verified to have correct institutional affiliation, 13 had positively identified errors, and no information was available about the accuracy of the final 15. Given that information is subject to change sporadically during a career, one less stringent concern is that information be accurate for researchers who are retired or no longer active in research. Of the 85 individuals identified outside of the database, 13 were no longer active, and 12 of these were accurately documented in Neurotree.

To assess how completely Neurotree represents the field, we also compared faculty rosters between Neurotree and three departmental web sites in institutions that varied in size and geography (Table 2). In all three cases, about two-thirds (63%) of departmental faculty were listed in Neurotree. Of those listed, 88% had correct institutional affiliation. Because departmental web sites are generally kept up to date, this analysis is likely to provide a reasonably accurate measure of representation of researchers in Neuroscience-oriented departments.

**Table 2:** Spot check of departmental representation in Neurotree for three departments varying in size and location.

Department	Count	Listed in Neurotree	Correct institution
Hebrew University, ICNC	27	22	20
Reed College, Psychology	9	6	6
University of Michigan, Neuroscience	114	67	58
Totals	150	95/150	84/95
Percent		63%	88%

Finally, we assessed how accurately and completely mentorship records were documented for five research groups by comparing trainee lists from public web pages and information in Neurotree (Table 3). For these groups, 75% of trainees listed on the web sites appeared in Neurotree. Of trainees listed in Neurotree, 30% did not appear on the lab websites. In all the cases studied, these mentor relationships could be verified by identifying at least one publication in Medline for which the mentor and trainee were co-authors. These results should be interpreted with caution. Because labs that maintain training records online might be more likely to also maintain Neurotree records, there may be a bias toward more complete representation of mentor relationships for these research groups. However, it is interesting to note that in many cases, Neurotree contained more up-to-date information than the most accurate alternative resource.

**Table 3:** Spot check of trainee listing accuracy for individual mentors.

Mentor	Institution	In lab web site	In Neurotree	In Neurotree, not lab site
C. Daniel Salzman	Columbia University	14	8	0
Patricia Kuhl	University of Washington	15	5	1
Barbara Chapman	University of California, Davis	7	8	2
Robert Malenka	Stanford University	15	25	14
Lynn Robertson	University of California, Berkeley	16	11	0
Totals		67	40/67	17/57
Percent			75%	30%

Lists of trainees (postdoctoral fellows only for Malenka, graduate students and postdoctoral fellows for all others) were compared between the websites of principal investigators who publish this information and mentorship data in Neurotree. In a few cases, Neurotree documented relationships that did not appear in the lab web sites (right column). All of these relationships were confirmed as accurate by a Medline publication record.

### Quantitative analysis of growth and connectivity

**Figure 5:**
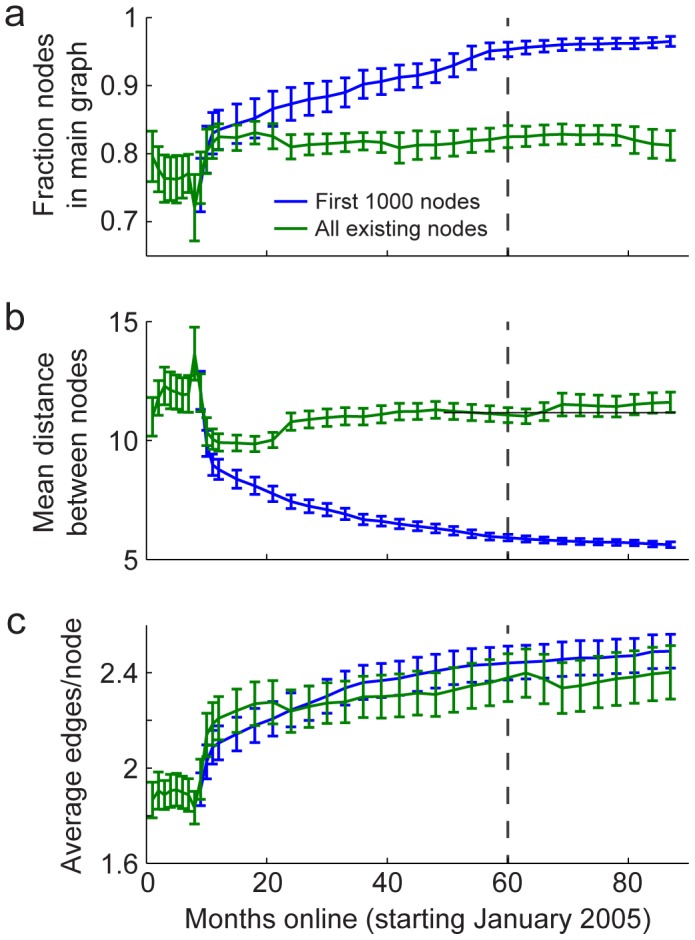
Basic connection properties of Neurotree. **A,** Fraction of researchers (nodes) connected to the main tree graph through mentor relationships, as a function of months since Neurotree was established. The blue line shows data for the subset of the first 1000 researchers added to the tree (starting in month 10, when the one-thousandth research was added) while the green line shows data for the entire tree. Error bars indicate one standard error on the mean, calculated by jackknifing. **B,** Mean distance between nodes over time, either within the subset of the first 1000 researchers or across the entire tree, plotted as in A. C, Average number of connections per node, plotted as in A.

For a more quantitative analysis of the maturity of Neurotree's connectivity, we measured on the temporal dynamics of three statistics: the fraction of researchers linked in the main graph (Figure 5A), the average distance between researchers (Figure 5B), and the average number of connections per researcher (Figure 5C). Because data about researchers added to the tree earlier are likely to be more complete than later entries, we compared these statistics for the first 1000 nodes entered into the tree and for the entire tree.

The trajectory of statistics for the first 1000 nodes follows a distinct pattern from that of the entire tree. The 1000th researcher was added when Neurotree had been online for 10 months. After that time, the fraction of these nodes connected to the main graph steadily increased until about month 60, at which point the fraction reached an asymptote of 96%. Simultaneously, the average distance between each pair of these nodes dropped and also reached an asymptote of 5.5 steps. The average number of connections per researcher to other researchers, finally, stabilized at 2.5. A slight ongoing rise in number of connections appears to reflect new connections that continue to form between members of the group. The fact that the number of connections within the first 1000 entries has stabilized does not mean that connections of these nodes with the rest of the tree have done the same. The average number of connections from this group to the entire tree has grown to 10, and continues to grown at a rate of 0.8 connection per year (data not shown). This ongoing growth likely reflects both the entry of new researchers into the field as well as the filling in of earlier connections.

In contrast to the first 1000 entries, we observed that the statistics of the full tree have remained more or less flat since the first year online. The fraction of nodes connected to the main graph has remained stable at about 80%. The mean distance between nodes has very slowly risen from 10 to 11 steps. Finally, the average number of connections per node has remained nearly constant at 2.2. This suggests a balance between the rate at which new entries are added and connections between older entries are filled in more completely.

### Identifying the founders of neuroscience

As illustrated by the example tree (Figure 1), individual researchers, through their trainees and the subsequent trainees of those trainees, can influence a large number of subsequent researchers. Measuring the fecundity of researchers according to their number of trainees provides a means of comparing their relative influence and determining which individuals have had the greatest influence on the field. Of course, fecundity is not a direct measure of influence, but rather a useful and readily quantifiable proxy.

We measured a fecundity index by counting iteratively the number of trainees and trainees of those trainees, normalized exponentially by the number of steps from the original mentor (see Methods, *γ* = 1/2). Normalization was critical to prevent attributing the most influence to the very earliest researchers, who would always have the most offspring in a non-normalized count (i,e., *γ* = 1). At the other extreme, a strong normalization factor (e.g., *γ*≤1/10) would place the most weight on the mentor's immediate trainees and would simply equate fecundity with large research groups.

The 25 researchers with the highest fecundity index appear in Table 4. On this list are a number of individuals typically associated with critical advances in the field. The majority of these individuals were active mentors (based on their first mentoring year, i.e., the first year a student of theirs was awarded a degree) between 1900 and 1950, though the remainder range broadly from 1842 (Johannes Müller) to 1974 (Torsten Wiesel).

**Table 4:** The founders of neuroscience, as ranked by fecundity measured from the Neurotree database (normalization factor, *γ* = 1/2, see Methods).

Rank (*γ* = 1/2)	Name	Institution	Year	Gen	Rank (Alt *γ*)		
					1	1/4	1/10
1.	John Eccles	Australian National University	1937	20	153	1	11
2.	Charles Sherrington	University of Oxford	1901	19	117	8	168
3.	Stephen Kuffler	Harvard University	1962	21	167	2	26
4.	Karl Lashley	Harvard University	1924	20	159	5	80
5.	John Langley	University of Cambridge	1900	19	113	111	849
6.	Michael Foster	University of Cambridge	1870	18	109	162	1342
7.	Edgar Adrian	University of Cambridge	1923	20	155	43	273
8.	Donald Hebb	McGill University	1952	21	199	9	58
9.	Robert Yerkes	Yale University	1918	19	154	105	645
10.	Johannes Müller	Humboldt Universität zu Berlin	1842	16	75	81	178
11.	Wilhelm Wundt	University of Leipzig	1886	17	111	106	206
12.	Bernard Katz	University College London	1952	21	204	19	82
13.	Torsten Wiesel	Rockefeller University	1974	22	267	4	14
14.	Keith Lucas	University of Cambridge	1904	19	136	236	1826
15.	Hans- Lukas Teuber	Mass. Inst. of Technology	1965	21	231	13	85
16.	John Black Johnston	University of Minnesota	1907	22	157	231	2077
17.	John Watson	Johns Hopkins University	1916	19	158	234	2095
18.	Clinton Woolsey	University of Wisconsin	1964	21	230	25	141
19.	Philip Bard	Johns Hopkins University	1928	20	197	77	701
20.	Hugo Munsterberg	Harvard University	1902	18	137	193	738
21.	John Fulton	Yale University	1932	20	168	62	255
22.	Wilder Penfield	McGill University	1952	20	181	91	535
23.	Hallowell Davis	Harvard University	1935	21	170	118	598
24.	Archibald Hill	University College London	1915	20	171	82	264
25.	Julius Axelrod	National Inst. of Mental Health	1962	22	255	12	44

Year refers to first year a degree was awarded to a trainee of this mentor. Generation (Gen) refers to the number mentorship steps back to the oldest common ancestor. Rankings for these individuals based on measures with alternative (Alt) normalization factors appear in the columns at right. At the extremes, *γ* = 1 weighs all offspring equally, regardless of generation, and *γ* = 1/10 counts primarily the number of direct trainees and gives very little weight to later generations.

To illustrate the importance of appropriate normalization in the fecundity calculation, Table 4 also lists rankings for these 25 individuals computed using different normalization factors in to compute fecundity. At the extreme of *γ* = 1 (no normalization), it is clear that rankings are much higher for researchers in earlier generations, as this metric simply gives highest rank to the earliest connected researchers. At the other extreme of *γ* = 1/10 (strong normalization), more recent researchers are ranked much higher, reflecting the recent trend toward larger research groups (at least as documented in Neurotree). The differences are not so extreme for a more modest adjustment, *γ* = 1/4. It is interesting to note, however, that even for this adjustment, historical figures who did not themselves have large groups but did train a small number of influential researchers (e.g., Michael Foster, Wilhelm Wundt) fall substantially in rank.

### Growth and development of the field of neuroscience

**Figure 6:**
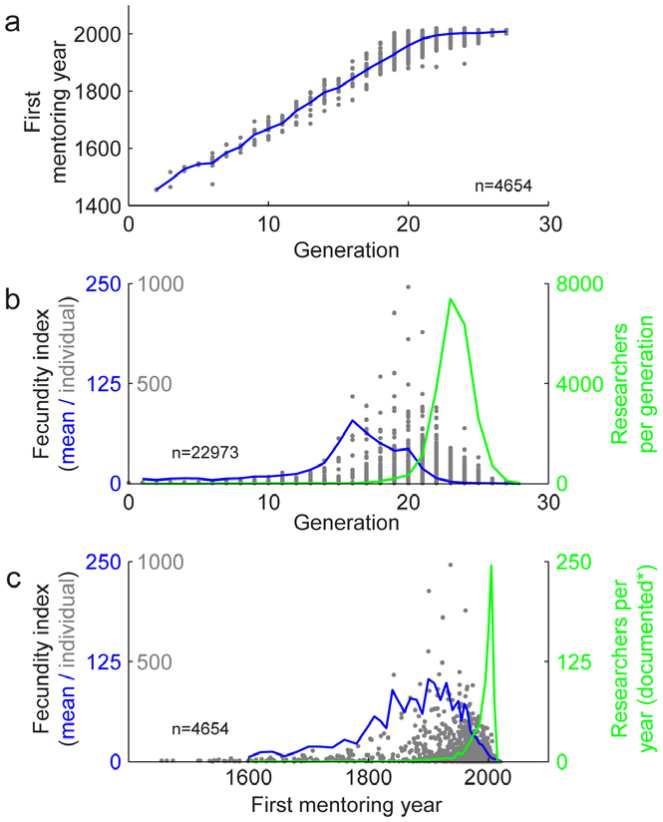
Historical influence of neuroscience researchers through mentorship. **A,** Comparison of researcher generation, the number of mentorship steps back to a common ancestor, versus the year the researcher began mentoring, for the subset of researchers for which this information is available. Gray dots indicate individuals and the blue curve indicates the average year for each generation (*r* = 0.88). **B,** Fecundity index (the number of descendents per researcher normalized exponentially by the number of steps from that researcher) as a function of generation. Gray dots and blue curve plotted as in A. Green curve indicates the number of researchers per generation. **C,** Fecundity index and average number of new researchers per year for the subset of nodes with mentorship year data, plotted as in B.

One challenge to precise interpretation of the temporal features of these data is that dates are not recorded for a substantial number of connections in Neurotree. In order to include a larger pool of researchers in the analysis of temporal dynamics, we computed the mentorship generation for each researcher by counting the number of steps back directly to their oldest ancestor. As discussed above, 64% of researchers in Neurotree can trace their mentorship back to a single individual. When we compared generation versus first mentoring year for the subset of researchers with appropriate data, we found a very strong correspondence (Figure 6A).

Using mentorship generation as a proxy measurement for time, we could then study the timecourse of fecundity across the field (Figure 6B). This analysis shows that average fecundity was greatest around generation 16, although the researchers with greatest fecundity tended to fall later, in generations 19–21. All these influential researchers largely predate the vast expansion of the field, which was just beginning in generation 21 (green line, Figure 6B).

To confirm that mentorship generation captures the essential features of a more strictly defined temporal analysis, we repeated the analysis of fecundity over time, but now focused only on the subset of 4654 researchers for which mentorship dates were available (Figure 6C). This analysis, while noisier, revealed similar trends. The period of greatest fecundity ranged from 1840–1950, with the most influential individuals appearing at the later end. The huge growth of the field can also be observed in the spike in the number of newly documented mentors that currently peaks for the period 2005–2010.

### Mentorship-based cluster analysis

**Figure 7:**
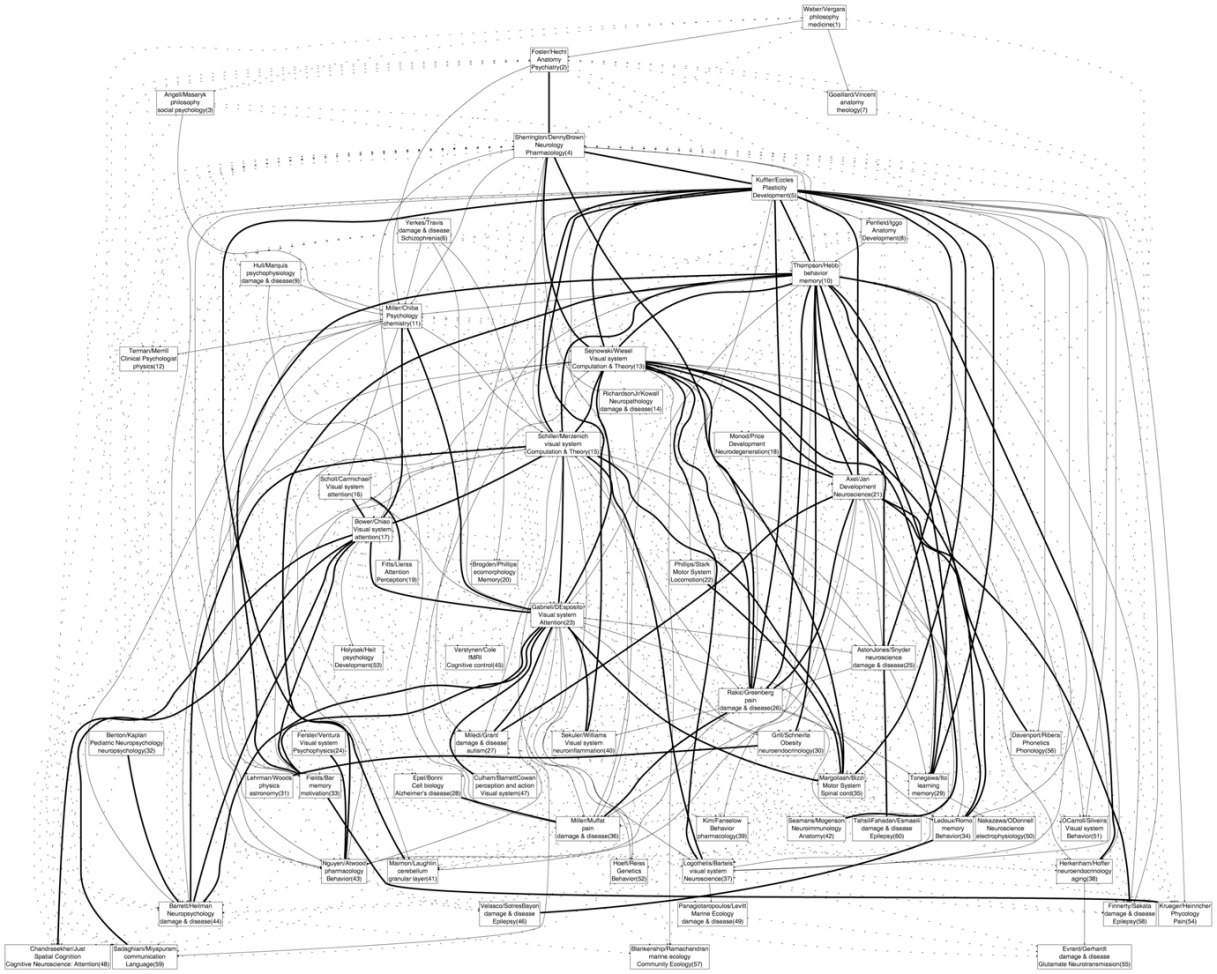
Mentorship-derived clusters. Clusters were derived by spectral factorization of the sparse matrix of mentor relationships between all researchers connected in the main Neurotree graph. Each box describes a cluster, numbered according to the average generation of researchers in the cluster. Clusters are plotted in roughly chronological order from top to bottom. Each cluster is labeled with the names of the two researchers with the smallest mean distance to other researchers in the cluster and by the two most common research areas in the cluster. Lines connecting clusters indicate the relative strength of connections between them (dotted: 1–5, solid: 6–20, bold: 21+).

In order to study the relationship between mentorship groups and research areas within neuroscience, the entire set of connected nodes (30055/35953 researchers) was clustered into 60 groups based on the strength of mentorship connections (see [6] and Methods). Results are plotted (Figure 7), sorted on the y-axis roughly by time (i.e., by the mean generation of researchers contained in each cluster). An interactive version of this analysis is available online at http://neurotree.org/neurotree/clusters.php [5].

Each cluster was labeled with the names of the two researchers with the lowest mean distance to other researchers in that cluster and by the two research areas most frequently occurring across the cluster. Despite being derived through independent metrics, research areas representing a cluster typically show an obvious relationship to the representative researchers. For example, in cluster 10, Donald Hebb and Richard Thompson are both associated with the study of memory. Likewise, for cluster 13, Terrence Sejnowski and Torsten Wiesel are associated with visual and systems neuroscience. The information in Neurotree about research areas is not complete, as it depends on unconstrained choices by users entering the data. This incomplete information could lead to some of the apparent discrepancies (e.g., cluster 26, Rakic and Greenberg are not immediately associated with pain research).

Some neighboring clusters identify logical divisions between research areas. For example, cluster 15 (Schiller/Merzenich) captures a number of researchers who study sensory processing in non-human primates while cluster 23 (Gabrieli/D'Esposito) includes researchers who study similar problems of representation in humans.

**Figure 8:**
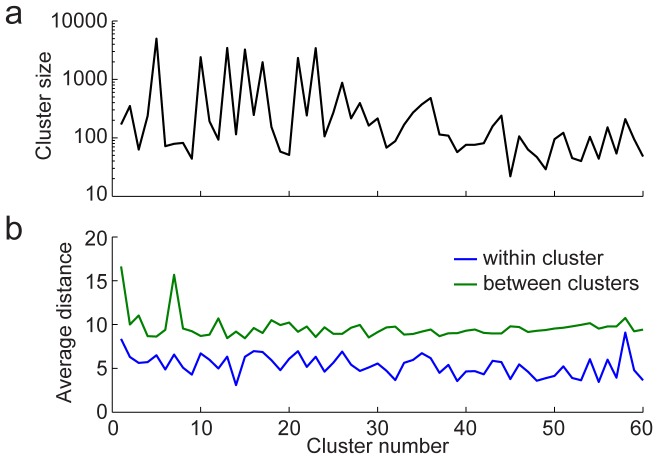
Quantitative analysis of mentorship clusters. **A,** Curve shows the number of researchers in clusters 1–60, plotted on a logarithmic axis. The very large clusters (>3000 researchers) suggest that some groups, which often appear centrally in Figure 6, are difficult to partition with the current algorithm. **B,** Average distance between nodes within each cluster (blue) and with nodes in other clusters (green). Small within-cluster distance and large between-cluster distance indicate groups that were well partitioned from the main Neurotree graph.

When the clusters were studied more quantitatively, a few additional features were noteworthy. First, the size of clusters varied substantially (Figure 8A). This distribution suggests that some subfields of neuroscience may partition off more easily than others. Very large clusters are problematic, as they encompass a large variety of work and may require more elaborate procedures in order to be segmented effectively.

A more informative analysis may be to study how tightly coupled clusters are, relative to their average distance from other clusters (Figure 8B). Small within-cluster distance and large between-cluster distance represent highly interconnected and easily partitioned groups. The most striking examples of these appear to be more clinically-oriented groups. For example, cluster 14 (mean distance within 3.1, between 9.2) focuses on neuropathology and cluster 32 (within 3.7, between 9.8) focuses on neuropsychology. Groups with large within-cluster distances can result from number of factors. Clusters 1 and 2 are likely to have large within-cluster distance simply because they contain researchers spread out over long historical periods.

## Discussion

Data documenting the tradition of academic mentorship naturally provoke curiosity to most people who have participated in the system. Each of us has received training from someone, who in turn was trained by someone else, and the whole process continues, iteratively, into the unknown past. An understanding of one's academic mentorship allows one to connect oneself to the historical development of a field. A genealogical tree also provides the opportunity to see otherwise invisible links between ourselves and our colleagues, our friends, and important figures in the field. For these reasons, academic genealogies have been created for many fields. Neurotree is an attempt to do so for the large and diverse field of neuroscience.

Good mentoring is a skill that can differentiate successful from unsuccessful lab leaders. As of yet, very little is known about how important mentorship skills are in producing successful progeny [4]. Although most experts rely on judgments informed by anecdotal evidence, it is difficult to separate out the effects of institution, age, and serendipity from individual skill. Neurotree provides quantitative data that can be used to develop more sophisticated understanding of the influence of mentorship on progeny success. This information can help identify the qualities that make good mentors, which can in turn be used to direct training of mentors, guide hiring decisions, and help students choose mentors.

More generally, Neurotree provides an important tool for the study of the birth, life, and death, of ideas. Central to the function of academic mentorship is the transmission of ideas from mentor to trainee. Thus having a clear and full database of individuals and their relationships can serve as a tool for studying the life cycle of ideas.

We argue that Neurotree has a specific role in the field of neuroscience. It provides a single repository for valuable information that is both highly specific and well-defined (such as mentor-trainee relationships) and that is more open-ended (such as field of interest). Additionally, Neurotree presents an opportunity to sort out potential confusion regarding multiple researchers with the same name. While there is no current widely accepted unique identifier for individual scientists, the Neurotree database can help discriminate among individuals.

### Maturing of Neurotree

As an experiment in crowd-sourcing the acquisition of data, Neurotree has been successful thus far. The Society for Neuroscience, whose academic focus encompasses a similar scope to that of Neurotree, lists 41,000 current members. This number does not include historical figures or neuroscientists who have not joined the Society, but the order of magnitude of this number matches that of the number of researchers listed in Neurotree. Given its record of growth, we expect Neurotree to develop a progressively more complete description of the field, thereby allowing reliable and unbiased sampling of mentorship relationships, and increasingly more accurate measures of progeny counts and connection distances. We have identified limitations to the scope of the dataset, both in its accuracy and completeness, and it remains an open question as to how completely these gaps can be filled with the current crowd-sourcing approach.

In addition to the general problem of sampling, crowd-sourcing efforts face the additional challenge of possible bias in how data is sampled [8]. Bias in gender, institution, geography, etc. can distort results in ways that are more difficult to correct than random sampling errors. Our initial analysis of accuracy revealed generally consistent levels of accuracy across institutions and labs, and Neurotree may benefit from the fact that a complete mentor-trainee record is well-defined. However, a more extensive analysis is required to determine if any systematic sampling bias exists.

Even with an incomplete data set, we have demonstrated approaches for approximating missing data from the database (e.g., using generation as a substitute for first year of mentoring). This has permitted us to include a much larger data set into the analysis of historically influential figures in the field.

### Potential for expanded scope and depth

Neurotree can serve a number of functions, all of which would be improved if data in the tree were more complete and accurate. Substantial data sources exist in the public sphere online that could be used to automatically or semi-automatically fill in gaps in institutional affiliation, mentorship dates, and research areas in the current database. These resources include structured databases (e.g., [9]) and less structured on-line content such as departmental web sites and the *CV*s of individual researchers.

Numerous additional data resources exist that can be incorporated into Neurotree. Information about the contents of publications (methods, preparations, scientific questions) can be linked to individual researchers, providing a means of systematically studying the relationship between mentorship and the experimental approaches adopted by trainees. As expanded scientific content is linked to researchers, Neurotree will provide an increasingly powerful tool for studying the evolution of the field.

### A complete, interdisciplinary academic genealogy

The software that forms the basis for Neurotree can be readily adjusted to make a database for any academic field. Based on unsolicited requests, we have created academic trees for other disciplines that, as far as we know, lack one of their own. These other trees include history, linguistics, and marine ecology, as well as a dozen others. Although they are given their own tree for display, they draw from the same database. This shared database permits cross-listing researchers between trees in different disciplines, so that, as the trees fill in, it will be possible to trace the larger-scale linkages between fields. Furthermore, it will be possible to study not only the graphical properties of mentor relationships within neuroscience but also how ideas and trends have traveled between fields.
